# Disparities Influencing Functional Outcomes Between Rural and Urban Patients With Acute Stroke

**DOI:** 10.3389/fneur.2022.869772

**Published:** 2022-05-09

**Authors:** Natalia Llanos-Leyton, Carlos Pardo, Gabriel D. Pinilla-Monsalve, Akemi Arango, Jaime Valderrama, Isabella Pugliese, Pablo Amaya

**Affiliations:** ^1^Faculty of Health Sciences, Universidad Icesi, Cali, Colombia; ^2^Stroke Clinic, Fundación Valle del Lili, Cali, Colombia; ^3^Clinical Research Center, Fundación Valle del Lili, Cali, Colombia

**Keywords:** ischemic stroke, health inequities, rural, urban, outcomes

## Abstract

**Introduction:**

There is scarce information in Latin America about factors related to stroke patient outcomes in rural areas compared to urban ones.

**Objective:**

To evaluate functional outcomes of stroke code patients from rural and urban areas and their relationship with socioeconomic disparity.

**Methods:**

Prospective cohort study included patients of urban, semi-urban, and rural origin with stroke code from a high complexity hospital in southwestern Colombia between 2018 and 2019. Demographic, clinical data modified Rankin at discharge, and 3-month follow-up were analyzed. The poverty index, barriers to health access and availability of ambulances by the municipality was assessed at an ecological level.

**Results:**

Five hundred and fifty five stroke patients were registered, 21.2% from rural areas, 432 (77.98%) had an ischemic stroke. There were no significant differences in sociodemographic factors and medical background. Urban patients had lower reperfusion therapies rates (23.25%). Favorable mRS at discharge (<3) was higher in urban areas (63.03%) and mortality was superior in rural patients (13.56%). The ambulance rate in semi-urban and rural areas was as low as 0.03 per 100.000 inhabitants, the poverty index was 11.9% in urban areas vs. 23.3% in semi urban and rural areas.

**Conclusions:**

Rural patients treated in our center were more likely to present with severe strokes and unfavorable mRS at hospital discharge and 3-month follow-up compared to urban, despite having similar risk factors. There is an inverse relationship, which is not related to the poverty rate or the percentage of people with barriers to access to health. There is a need for further studies that assess barriers inherent in rural patients and establish a regional stroke network.

## Introduction

In high-income countries, stroke mortality has decreased during recent years using treatments such as systemic thrombolysis and mechanical thrombectomy. However, there are some population groups in which lethality could be significantly higher. Patients from rural areas seem to suffer more severe strokes, although this relationship could be moderated by a higher incidence rate (1). In particular, more than 600,000 stroke patients per year in the United States do not receive adequate treatment in rural areas, mainly due to longer times to obtain a specialized neurological evaluation which implies an increase in mortality of up to 20% compared to that registered in patients from large cities (1).

According to the ESENCIA study, in 2017, there was a prevalence of 158 (CI 95% 157-160) cases per 100,000 inhabitants of ischemic stroke in Colombia. The prevalence was higher in patients affiliated to the contributory insurance regime (financed by employee-employer contributions) (62.11%, OR 1.488, 95% CI 1.454-1.523, *p* < 0.001). The highest stroke prevalence was registered in the regions of Bogotá (438, 431-444), Cundinamarca (407, 396-419), and Chocó (312, 277-350) (2). According to the Global Burden of Disease, ischemic stroke in Colombia caused 8,295 deaths (6,266-10,477) and an absolute number of 137,162 ^*^DALYs (11,328-166,980) in 2019 (3).

There is evidence that the most critical disparity in Latin America is related to the differential access to medical services among rural and urban patients, which leads to biased epidemiology information and an underestimation of the current public health problem (4). The prehospital care for stroke in low and middle-income countries (LMICs) is underdeveloped, and there is a poor pre-notification system associated with longer door-to-image, door-to-needle, and door-to-groin times and, consequently, worse prognosis (5). Pre-hospital recognition of a neurovascular syndrome is key as the rates of intravenous thrombolysis could rise from 23 to 42% (4).

In Colombia, only 13 cities out of the 1,103 municipalities have thrombectomy centers and, of the 34 hospitals enabled to perform this intervention, only 14 provide 24/7 services (6). According to the World Health Organization (WHO), a city should have one ambulance for every 25.000 inhabitants (7) to improve and guarantee pre-hospital care.

Given the high prevalence of the diseases and the lack of knowledge about the geographical barriers in acute stroke patients accessing health services, it is necessary to clarify how these characteristics are associated with their clinical outcomes. Therefore, this study aims to compare the pre- and intra-hospital care received by acute stroke patients from rural, semi-urban, and urban areas treated in a comprehensive stroke center located in southwestern Colombia and determine potential differences in outcomes related to patients' precedence.

## Methods

This is a prospective cohort study of acute stroke patients based on demographic and clinical data collected between January 2018 and January 2020 who were admitted in a high complexity hospital from Southwestern Colombia as part of a pilot stroke network, consisting of rural primary centers without the capacity of imaging or thrombolysis and a mothership hospital.

The included subjects were patients over 18 years old who attended Hospital Universitario Fundación Valle del Lili within a stroke code context. Confirmation of ischemic and hemorrhagic strokes was based on computer tomography or magnetic resonance imaging, and a vascular or trained general neurologist diagnosed transient ischemic attacks (TIA) according to the clinical manifestations and current guidelines. We excluded patients who underwent extra-institutional reperfusion therapy, those without institutional diagnostic images, stroke mimics, or those who presented the cerebrovascular event during hospitalization. We considered each stroke code consultation as a separate registry.

Mode of arrival at the stroke center, timing, clinical characteristics, interventions, and modified Rankin scale (mRS) for ischemic stroke patients at onset, discharge, and 3-months follow-up were assessed. mRS was considered favorable if <3. Distance from rural and semi-urban facilities to the mothership center was extracted from Google Maps (8) and generally set as 11 Km for urban patients (half of the city's maximum extension).

In Colombia, according to the *Departamento Administrativo Nacional de Estad*í*stica* (DANE), a metropolitan area is defined as the administrative entity formed by a set of two or more municipalities. The metropolitan area of Cali comprises Palmira, Yumbo, Jamundí y Candelaria (semi-urban municipalities). Urban areas are limited to the set of buildings and blocks that configures adjacent group structures. On the contrary, the rural area has a dispersed disposition of houses and farms, without nomenclature of streets (9).

Ecological variables of municipalities (total population, poverty index, percentage of inhabitants with evident healthcare barriers, and the number of total, primary, and medicalized ambulances) were extracted from their most recent Health Situation Analysis (ASIS, 2017-2020) and compared with the change in mRS between onset and discharge/3-months follow-up (primary outcome) and total length of in-hospital and ICU stay.

### Statistical Analysis

According to their distribution, qualitative variables were described with absolute and relative frequencies and numerical features with medians and interquartile ranges. Differences between urban, semi-urban, and rural areas were analyzed using multiple comparisons χ^2^ test and Kruskal-Wallis statistic with Dunn's *post-hoc* method and Sidak correction (*p*-values reported for rural vs. urban only). Wilcoxon test was implemented for identifying significant changes between mRS at discharge/3-months follow-up and onset.

Poisson multivariate regression models were built using the change in mRS between onset and discharge/3-months follow-up as the primary outcome and selecting independent variables through a stepwise backward method. Different models were defined for total, rural, and urban code-stroke patients. An additional logistic model was proposed for differentiating rural and urban populations after excluding patients from semi-urban areas. The odds ratio and 95% confidence intervals were calculated. Correlations of ecological variables of municipalities with the outcomes were assessed using Spearman's rank coefficient. Significance was set as *p* < 0.05. Statistical analysis was performed in Stata v.15.0 (StataCorp, College Station, TX, USA).

Institutional review board approval was granted for this study as part of the continuous prospective registry of stroke patients (No. 422/2019). An abstract of a preliminary analysis was recently published (10).

## Results

### Patients and Neurovascular Events Features at Arrival

All the 555 code-stroke patients who attended the emergency department were included in the study: 357 (64.3%) from the urban area, 80 (14.4%) from semi-urban locations, and 118 (21.2%) from rural sites. Baseline mRS was favorable (0-2) in the majority of patients from urban areas (91.92%) semi-urban (89.18%), and rural (88.8%).

According to the geographical origin, there were no significant differences in age, gender, or medical background, except for previous TIA, which was more common in urban patients 7.84% vs. semiurban 0.00% vs. rural 5.08%; global *p* = 0.0260, *post-hoc p* = 0.3140. In detail, rural and semi-urban individuals had a similar absolute prevalence of arterial hypertension; however, the frequency of atrial fibrillation, dyslipidemia, and previous myocardial infarction was numerically higher in urban subjects (Table 1). Additionally, rural patients reported mildly lower use of statins (23.73%) than semi-urban (32.50%) and urban areas (31.93%). Semi-urban patients tended to be prescribed anticoagulants more frequently than urban and rural individuals, but the intake of antiplatelet agents was equivalent among all groups.

**Table 1:** Baseline characteristics of patients.

**Characteristic**	**No (%)**			
	**Urban**	**Semi-urban**	**Rural**	***P*-value**
	**(*N* = 357)**	**(*N* = 80)**	**(*N* = 118)**	
Median age (IQR), years	70 (60-81)	71 (61-80)	71 (60-81)	0.9324
Female	192 (53.78)	37 (46.25)	56 (47.46)	0.3020
BMI (Kg/m2) IQR	26.5 (23.9-29.1)	25.4 (23.1-28.4)	27 (25-29.4)	0.363
Arterial hypertension	243 (68.07)	65 (81.25)	86 (72.88)	0.0560
Diabetes mellitus	86 (24.09)	20 (25)	31 (26.27)	0.8900
Atrial fibrillation	34 (9.52)	9 (11.25)	10 (8.47)	0.8080
Previous smoking	15 (4.2)	3 (3.75)	4 (3.39)	0.9210
Current smoking	30 (8.4)	8 (10)	12 (10.17)	0.7990
Dyslipidemia	45 (12.61)	6 (7.5)	7 (5.93)	0.0790
Myocardial infarction	64 (17.93)	12 (15)	17 (14.41)	0.6080
Deep venous thrombosis	4 (1.12)	1 (1.25)	3 (2.54)	0.5260
Prior ischaemic stroke	67 (18.77)	11 (13.75)	25 (21.19)	0.4120
Prior hemorrhagic stroke	8 (2.24)	0 (0)	0 (0)	0.1050
Prior transitory ischemic attack	28 (7.84)	0 (0)	6 (5.08)	0.0260
mRs premorbid (IQR)	0 (0-1)	0 (0-1)	0 (0-1)	0.9266

*BMI, body mass index; AF, atrial fibrillation; mRs, modified rankin scale*.

Symptoms commonly started between 8:00 and 15:50 h (46.37%). Urban patients (38.74%) were less likely to be transported by ambulance than those from semi-urban (83.54%) and rural areas (92.31%). The median distance to the mothership hospital was 14 Km (IQR 14-14) for semi-urban subjects and 45 Km (IQR 45-53) for rural patients. Consequently, there was a higher median time window: for the urban area 230 min (IQR 1487.50-417.5) 165 min for semi-urban locations (IQR 89-314), and 151min (IQR 80-328) for rural sites (global *p* = 0.0001, *post-hoc p* < 0.0000).

### Clinical Characteristics During the In-hospital Stay

Rural (98.30%) and semi-urban (95%) patients were more likely to be classified as triage 1 and 2 (global *p* = 0.0010, *post-hoc p* = 0.000). Urban patients (18%) less frequently presented NIHSS scores compatible with a moderate-severe stroke (≥16) compared with semiurban (30%) and rural subjects (43%).

Door-to-image time was higher for urban patients (30 min, IQR 15-78) in comparison to semiurban (20 min, IQR 11-37) and rural subjects (22 min, IQR 12-40). Four hundred and thirty two (77.98%) patients had an ischemic stroke, with no significant differences according to patients' origin. The median score on the ASPECTS scale was lower in rural patients (8, IQR 7-10), while semi-urban (9, IQR 8-10) and urban (9, IQR 8-10) subjects exhibited a less extensive compromise (global *p* = 0.0114, *post-hoc p* = 0.0093).

There were significant global differences (*p* = 0.0021, *post-hoc p* = 0.0830) regarding the chosen treatment. In particular, as the percentage of ischemic stroke patients with an adequate window time (<4.5 h) was higher in semi-urban (68.75 %) patients compared with patients from urban (60.22 %) and rural areas (52.14 %), the use of intravenous thrombolysis (alone or combined with thrombectomy) was also higher in the semi-urban group (32.5%). Rural patients were treated more often with thrombectomy (alone or combined) (17.8%) (Table 2).

**Table 2:** Acute ischemic stroke treatment.

	**Urban**	**Semi-urban**	**Rural**	**Global *P*-value**	***Post-hoc P***
	**(*N* = 356)**	**(*N* = 80)**	**(*N* = 118 )**		
**Time window**					
<4.5 h	215 (60.22)	55 (68.75)	61 (52.14)	0.062	
>24 h	31 (8.69)	7 (8.75)	11 (9.4)	0.972	
Arrival by ambulance	117 (38.74)	66 (83.54)	108 (92.31)	0.000	
Door-to-image (IQR)	30 (15-78)	20 (11-37)	22 (12-40)	0.0004	0.0053
ASPECTS (IQR)	9 (8-10)	9 (8-10)	8 (7-10)	0.0114	0.0093
Medical treatment	274 (76.75)	52 (65)	80 (67.8)		
Thrombolysis	50 (14.01)	21 (26.25)	17 (14.41)		
Thrombectomy	14 (3.92)	2 (2.5)	9 (7.63)		
Combined	19 (5.32)	5 (6.25)	12 (10.17)		
Door-to-needle (IQR)	60 (47-85)	55 (45-81)	57 (45-70)	0.5486	
Door-to-groin (IQR)	132 (90-203)	116 (85-160)	157.5 (116.5-183)	0.6363	
In-hospital stay (days)	4 (2-9)	6 (2-12)	4 (2-11)	0.4927	
ICU stay (days)	3 (2-5)	3 (2-5)	3 (2-6)	0.8342	

*IQR, interquartile range; ICU, intensive care unit*.

Conservative management was offered to patients with lower NIHSS (5, IQR 2-12 vs. 12, IQR 8-20; *p* = 0.000), and the best empirical cut-point for deciding reperfusion therapy was found at 16.5. Subsequently, median door-needle time was higher in urban patients, differing from rural subjects (global *p* = 0.054). There were no relevant differences related to the door-groin time (*p* = 0.6363).

There were no significant differences between the groups in the length of in-hospital and ICU stay.

### Differential Functional Outcomes by Patients' Origin

**Figure 1:**
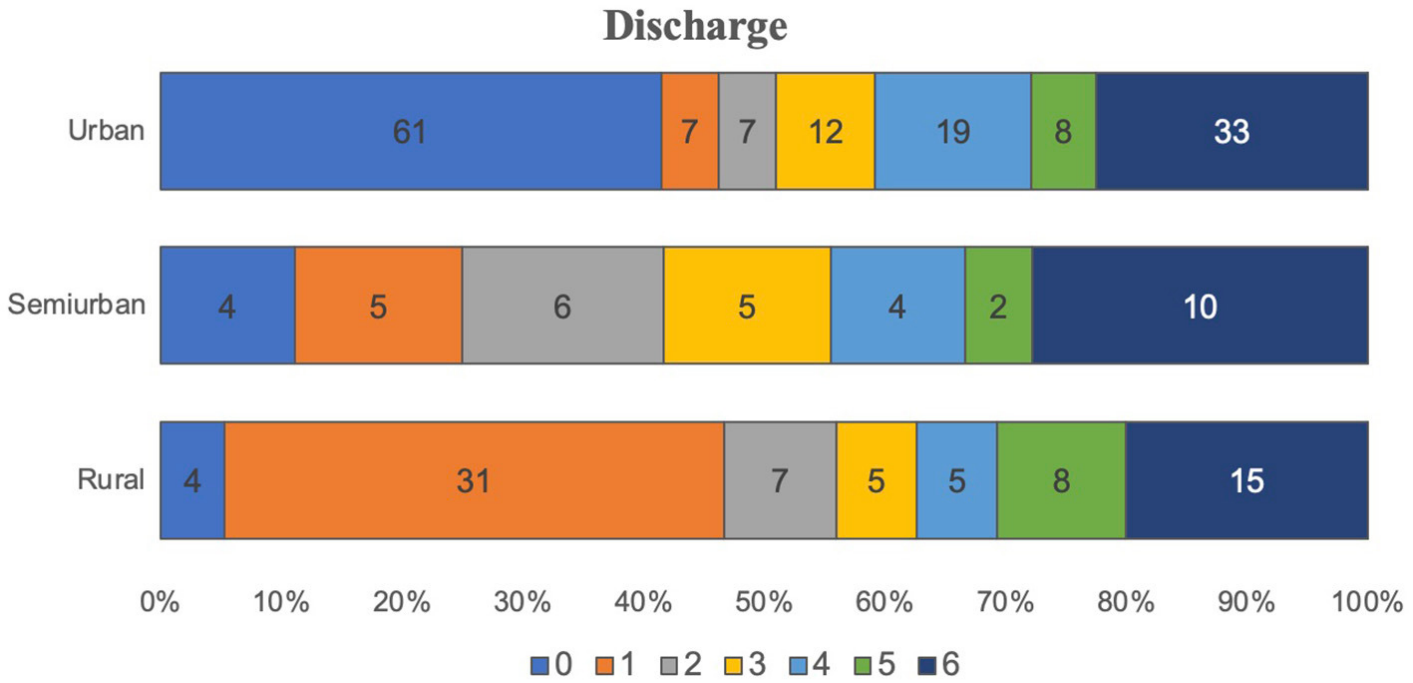
mRs scale at discharge.

**Figure 2:**
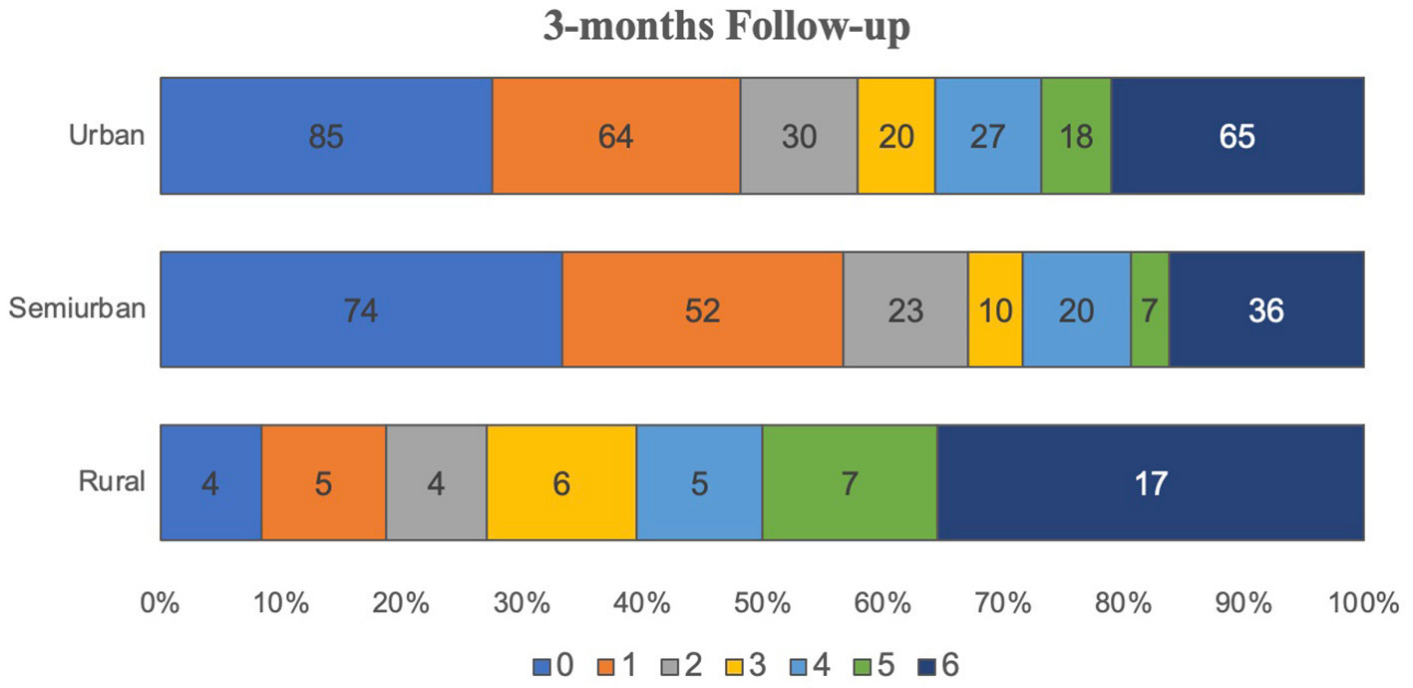
mRs scale at 3-months follow up.

Three hundred and thirty seven patients could be contacted to assess the telephonic rankin follow-up, among them 68.06% urban, 52.5% semi-urban, and 44.06% rural patients. Quantitative evaluation of mRS at discharge and 3-months follow-up showed significant differences (global *p* = 0.0001, *post-hoc p* = 0.0002). The percentage of patients with favorable mRS at discharge was higher in urban areas (63.03%) than in semi-urban (48.75%) and rural areas (31.35%) (global *p* = 0.0000, *post-hoc p* = 0.0000). Lethality (mRS 6) was higher in rural (13.56%) and semi-urban individuals (15%) compared with urban patients (10.64%) (Figures 1, 2).

Multivariate association model for higher mRS increase at discharge and 3-months follow-up in the whole ischemic stroke sample can be accessed in the Supplementary File. Higher NIHSS scores were associated with a worse decline in functionality for rural and urban patients at both time points. In subjects living in the city, previous myocardial infarction, more extended in-hospital stay, and receiving bridging therapy were also more common in those with poorer outcomes at discharge. Initial blood glucose levels and previous smoking showed similar associations at 3-months follow-up. Finally, patients' origin was associated with mRS variation at 3-months follow-up after multivariate adjustment by blood systolic and medium pressures, respiratory rate, use of statins, and triage classification (Supplementary File).

### Ecological Correlation of Municipality Conditions With Outcomes

Cali's urban area has a lower poverty index (11.9%) compared with semi-urban and rural populations (23.33%, IQR 15.68-63.87) and a higher number of ambulances (0.15 vs. as low as 0.03 per 100.000 inhabitants). There are 363 ambulances (11) in Cali for 2,496,442 inhabitants (12), leaving one ambulance for every 6,877 inhabitants. The ambulance rate in semi-urban and rural areas could be as low as 0.03. Patients from the city exhibited an mRS increase at the discharge of 1 point (IQR 0-3) vs. 3 points (IQR 2-3.5) in rural subjects. For instance, six patients from Buenos Aires (Cauca), a rural municipality located 53 Km from the mothership hospital, demonstrated a 3.5 points increase between baseline and discharge mRS. This municipality has a high prevalence of poverty (82.28%) and healthcare access barriers (43.83%).

**Figure 3:**
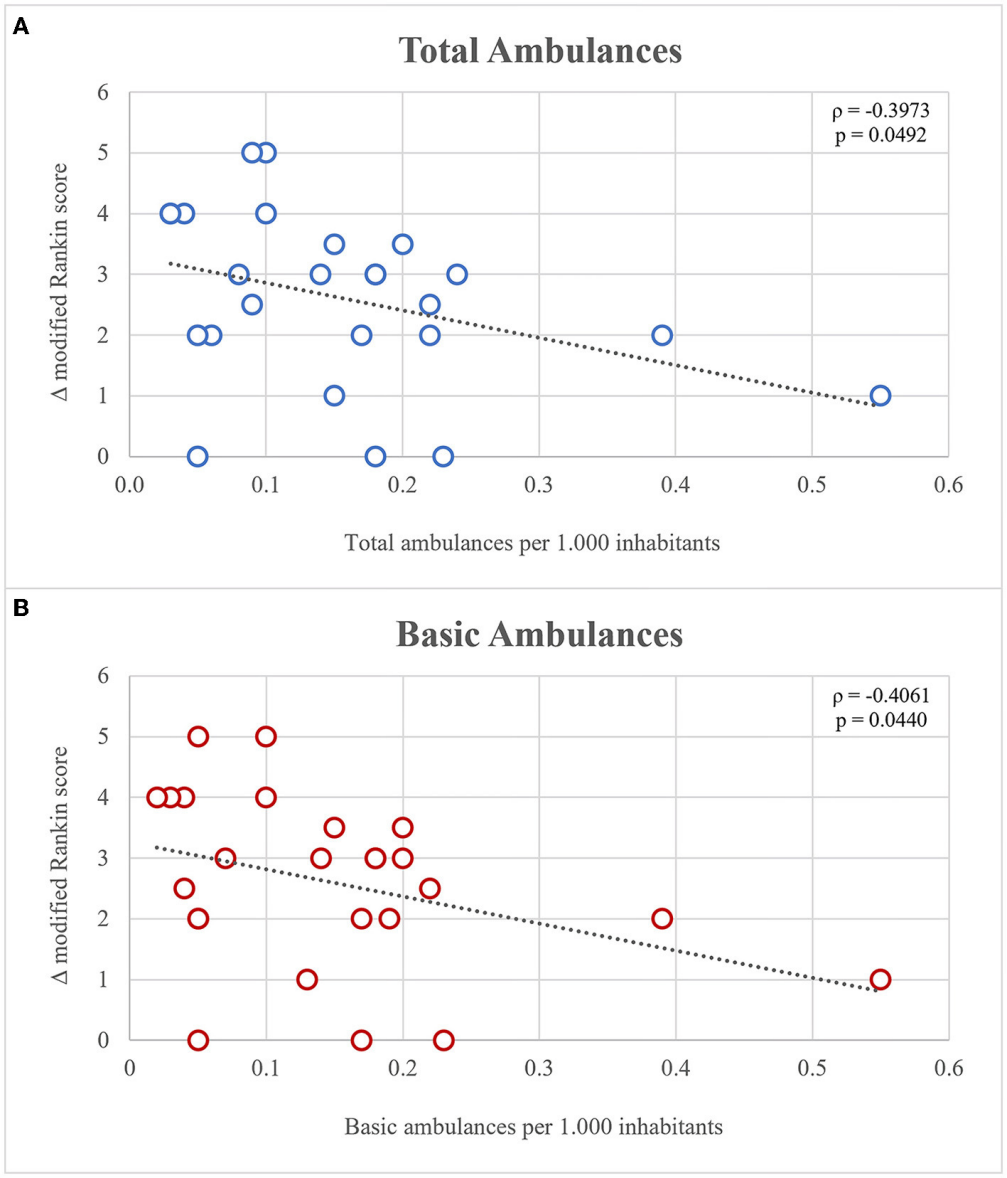
**(A,B)** Correlation of ambulance index and mRs and discharge.

The studied municipalities adhered to the WHO recommendations regarding the number of ambulances, except from three rural populations (Buenaventura, Guapi, and Corinto) located at 50-175 Km from Cali. Total (ρ = −0.3973, *p* = 0.0492) and basic (ρ = −0.4061, *p* = 0.0440) ambulance indexes were inversely correlated with the increase of mRS at discharge and 3 months follow-up (Figure 3).

## Discussion

To our knowledge, this is an innovative prospective study in Latin America comparing the pre-hospital and intra-hospital care of code-stroke patients, characterizing and differentiating their clinical outcomes based on their origin (rural, semi-urban or urban areas).

Patients from rural Southwestern Colombia were more likely to present severe stroke despite the registry of lower cardiovascular risk factors. Once arrived at the stroke center, the final diagnosis was reached faster, but disability was higher at discharge and 3-months follow-up. In agreement with previous studies (13–15), age and gender were similar among the three areas, and overweight, hypertension, diabetes mellitus were the most prevalent modifiable stroke risk factors. A previous history of ischemic stroke was more prevalent in rural patients, which increases the risk of a new stroke (16). Studies have shown that cumulative risk of stroke recurrence can vary from 7 to 20% in up to 5-year follow-up (17). History of myocardial infarction and dyslipidemia was higher in urban patients, probably because of an undiagnosed and underreporting of dyslipidemia in rural patients. Previous TIA was less prevalent in rural patients than the other groups, perhaps because they do not present to the emergency department when experiencing minor symptoms (18). Most of the subjects were overweight, mainly rural patients, who also had less prescription of statins; this is contradictory with other studies in which overweight is more prevalent in urban patients (19, 20).

In our study, patients had favorable mRs at baseline (global mRS median 0 (0-1). Patients from rural areas had higher window time than semi-urban and urban patients. This can be associated with longer distances and a lower education level about stroke symptoms, which is catastrophic in a pathology where treatment depends on timing (21). Consequently, these patients were more likely to have more severe strokes with higher NIHSS scores and lower ASPECTS, consistent with Kapral et al. study (22).

A higher percentage of rural patients arrived by ambulance outside the traditional and extended therapeutic window. In our study, only 38% of urban patients arrived by ambulance while other research studies have evidenced that urban patients are most likely to use this mode of transport within the therapeutic window (1, 22). This result is likely to occur because patients believe that there is someone else is greater need or unaware of the urgency (22). Also, literature reported the lack of public awareness about the need for an ambulance and the common perception of patients that a taxi or private car is faster than an ambulance. In a low-middle income country such as Colombia, there is an inefficient emergency medical service (EMS) in the cities, probably due to a lack of funding, roads, and traffic volume (23). Upon arrival at the stroke center, the diagnosis was reached faster for rural patients because their primary health centers usually refer them to confirm a severe stroke, making it easy to suspect the pathology (24). Even though, disability was higher at discharge and 3-months follow-up.

At the hospital, a non-contrast CT scan was the chosen image for most of the patients. Urban patients had longer window-times probably due to lack of pre-notification and less severe strokes (TIA). The most affected vessel in ischemic stroke was the MCA, according to the literature (5). Thrombectomy was more likely to be done in rural patients because of their longer window times, excluding patients to receive intravenous thrombolysis. Urban and semi-urban patients were more likely to receive rt-PA treatment than rural patients, which is consistent with the literature (25, 26).

Patients who lived in towns with a low ambulance index had worse mRs at discharge. This could be explained by differences in the opportunity for timely transportation, treatment, and rehabilitation, being worse in rural patients, and this might deleteriously impact their functional results. According to other studies, a rural residence has also been associated with a higher mRS and mortality rate (27).

Regarding the in-hospital stay observed in our study, there were no differences with the data reported by other authors (28, 29). Lethality rates were higher in rural and semi-urban patients compared to urban's. We suggest this happened because of patients' difficulties in identifying symptoms and delays in the ambulance services which are inefficient when pursuing reperfusion therapy (24).

### Related Factors With Functional Outcomes at 3-Months Follow-Up

In the multivariate association model, blood glucose levels, heart rate, and blood pressure were associated with mRS variation at 3 months follow up, which correlates with the literature. Autonomic nervous system dysfunction occurs after acute stroke and correlates with stroke severity and outcomes through increased blood pressure variability, impaired cerebral autoregulation, hyperglycemia, and blood-brain barrier dysfunction (30).

Non-urban patients who arrived by ambulance had higher mRs scores at discharge and 3 months follow-up than patients who arrived by private transportation due to more severe stroke. The unfavorable mRS score at 3-months follow-up could be due to fewer possibilities to obtain physical rehabilitation and other post-stroke services; this is clearly inadequate and far below evidence-based standards (31).

Although the significance is borderline, we observed that the municipalities with a fewer rate of ambulances demonstrated a higher increase in mRS at discharge (loss of functionality). An inverse relationship is not explained by the poverty rate or the percentage of people with healthcare access barriers. In that sense, the public administrations must create a pre-hospital care protocol and increase the ratio of ambulances per 1.000 habitants.

These findings support the need for further studies to understand additional variables that may contribute to the mortality of rural stroke patients. Our findings are likely to generalize to Colombia because of a similar demographic distribution where larger cities are surrounded by many small municipalities (semi-urban and rural areas). Patients are often referred from these rural territories to anywhere with a high-complexity healthcare center. Thus, the strategy of the godfather/mothership hospital can be strengthened to improve the detection and treatment rates of stroke patients, using telemedicine tools in a stroke network, generating better clinical outcomes.

### Strengths and Limitations

The sample of this study is small compared to international studies. As far as we know, this type of study has not been carried out in Latin America; however, studies from the region describe the prevalence and clinical outcomes of rural populations with stroke without comparing them with their urban counterparts (32). We also found studies comparing rural with urban populations, but these were carried out by applying questionnaires and did not consider treatment variables (33). We consider that the number of patients is adequate for the results obtained from this first experience.

Given the type of study, certain limitations should be mentioned. First, the collection of prospective information could have been biased after the creation of the institutional code-stroke and the standardization of treatments. Second, an important limitation comprises the loss in the follow-up of patients due to changes in their telephone number or because they did not answer. However, the follow-up rate was high. Another significant limitation was that the study was conducted in a single center in southwestern Colombia because we are starting a pilot program to create the stroke network. We need to create a local registry that includes all stroke centers to provide information about the incidence and prevalence of stroke in the city and the rural areas. Another limitation is the lack of data in rural hospitals about stroke cases.

In general, patients from rural areas had lower rates of dyslipidemia, taking into account that not all patients underwent a lipid profile, which could generate underdiagnosis and information biases. Rural patients presented more severe strokes compared to semi-urban and urban. However, we considered that it could be partly explained since the referred patients have a longer time window in the worst conditions. These patients also had worse functional outcomes at 3 months. There might be a smaller opportunity for access to post-stroke services considering that the probability is markedly influenced by the distance between healthcare centers and patients' houses and the state of the roads (1). These factors could not be studied as they are difficult to estimate from the perspective of the mothership hospital.

### Relevance to Our Region

Considering the findings obtained and the limitations presented in the study, we plan to develop strategies that could improve the disparities in care according to the origin of the patients. Our University hospital has been a pioneer in the region and the country by sponsoring rural hospitals through educational programs for fighting the lack of awareness of the disease. We also have promoted the activation of code-stroke by rural physicians who received personalized advice by a neurologist. We firmly believe that many rural patients could benefit from this sponsorship program by obtaining faster stroke care that results in better clinical outcomes. We need to work in an urban EMS program to increase the number of patients arriving in ambulance.

According to our institutional data, 45% of code-stroke patients come from rural areas. The study of differential clinical factors could favor the future design of strategies with a positive impact on prevention and early recognition of the disease, effective/comprehensive treatment, and the reduction of mortality and long-term disability.

## Conclusions

There is a lack of information in Latin America about the clinical outcomes of stroke patients according to their place of origin. We found a low percentage of patients from urban areas transferred by ambulance, needing to reinforce a pre-hospital care system in the city. Patients from rural areas of Southwestern Colombia were more likely to present with severe strokes and exhibited unfavorable mRS at hospital discharge and 3-month follow-up compared to their urban counterparts, despite having similar risk factors. Further studies should specifically assess the existence of barriers inherent to the place of origin, such as socioeconomic status, healthcare access, limited public transportation, inadequate pre-hospital emergency services, difficulties in accessing rehabilitation therapies, little knowledge of stroke symptoms, among others. Establish a regional stroke network could help to increase the access of the patients to a comprehensive stroke center.

## Data Availability Statement

The raw data supporting the conclusions of this article will be made available by the authors, without undue reservation.

## Author Contributions

PA, NL-L, and CP: study conception and design. PA, AA, JV, NL-L, CP, and GP-M: data collection. GP-M, PA, NL-L, and CP: analysis and interpretation of results. PA, NL-L, CP, GP-M, AA, JV, and IP: draft manuscript preparation. All authors reviewed the results and approved the final version of the manuscript.

## Conflict of Interest

The authors declare that the research was conducted in the absence of any commercial or financial relationships that could be construed as a potential conflict of interest.

## Publisher's Note

All claims expressed in this article are solely those of the authors and do not necessarily represent those of their affiliated organizations, or those of the publisher, the editors and the reviewers. Any product that may be evaluated in this article, or claim that may be made by its manufacturer, is not guaranteed or endorsed by the publisher.
