# Supplementary material for: Disparities Influencing Functional Outcomes Between Rural and Urban Patients With Acute Stroke
Source: Front Neurol. 2022 May 9;13:869772. doi: 10.3389/fneur.2022.869772 (PMC9124848; doi:10.3389/fneur.2022.869772)
Supplement: Supplementary file 1 [file Table_1.docx]

**Supplementary data 1 - Multivariate association model for higher mRS increase at discharge and 3-months follow-up**

| **Differences** | **Odds Ratio** | **Std. Err.** | **z** | **p>z** | **Lower CI** | **Upper CI** |
| --- | --- | --- | --- | --- | --- | --- |
| ∆ mRS 2 | 1.4659 | 0.1478 | 3.7900 | 0.0000 | 1.2031 | 1.7862 |
| Systolic pressure | 1.0421 | 0.0155 | 2.7800 | 0.0050 | 1.0122 | 1.0729 |
| Medium pressure | 0.9369 | 0.0202 | -3.0200 | 0.0030 | 0.8981 | 0.9774 |
| Respiratory rate | 1.1713 | 0.0811 | 2.2800 | 0.0220 | 1.0227 | 1.3414 |
| Statines | 0.2449 | 0.1349 | -2.5500 | 0.0110 | 0.0832 | 0.7207 |
| Triage | 0.2276 | 0.1234 | -2.7300 | 0.0060 | 0.0786 | 0.6589 |
| Constant | 0.0740 | 0.1437 | -1.3400 | 0.1800 | 0.0016 | 3.3317 |

∆ mRS 2: Difference between mRs at the 3 months follow up Vs discharge
